# The effectiveness of guided botulinum toxin “A” injection to the masticatory muscles in the management of condylar and sub condylar fractures: a randomized controlled clinical trial

**DOI:** 10.1186/s12903-026-09906-y

**Published:** 2026-10-12

**Authors:** Pakinam H. Gheeta, Adham A. Elashwah, Passent A. Younis

**Affiliations:** Department of Oral and Maxillofacial Surgery, Faculty of Dentistry, Alexandria University, Champlion Street, El-Azarita, Alexandria, Egypt

**Keywords:** Subcondylar fractures, Ultrasound, Botulinum toxin, Lateral pterygoid, Masseter, Temporalis, Closed reduction

## Abstract

**Objective:**

To evaluate the effectiveness of ultrasound guided injection of botulinum toxin A into the muscles of mastication; the masseter, temporalis and the lateral pterygoid, in a closed reduction approach of condylar and sub-condylar fractures, to optimize fracture reduction, reduce the length of intermaxillary fixation (IMF) and to facilitate postoperative functional recovery.

**Materials and methods:**

Sixteen medically healthy patients aged 18–35 years, presenting with unilateral subcondylar fracture were randomly assigned into two groups. The randomized controlled clinical trial was completed after recruitment, intervention, and follow up of all participants. The Study group (A) underwent an intervention involving ultrasound-guided Botulinum Toxin (BTX) injection into the Lateral Pterygoid Muscle (LPM), the Temporalis and the Masseter muscles, followed by the closed reduction treatment of the fracture, compared to the control group (B) that underwent conventional treatment method; the closed reduction approach without any BTX injections.

**Results:**

Pain scores were significantly lower in the study group (A), especially after the botulinum toxin injection (*p* < 0.001*) which subsequently led to a significantly shorter operation time (*p* < 0.001*) in the study group compared with the control group (B). Following surgery, mouth opening improved significantly over time in both groups (*p*** < **0.001*); however, the study group showed significantly greater improvement at 14 days (31.44 ± 2.72 mm versus 14.46 ± 3.24 mm, in the control group; *p* < 0.001*) and at 6 weeks postoperatively, (38.02 ± 3.26 mm versus 17.88 ± 4.49 mm; *p* < 0.001*) respectively. Regarding structural outcomes, patients in the study group demonstrated significantly better postoperative reduction accuracy, with lower angulation values (*p* < 0.02*) immediately postoperative and at 6 months compared with the control group. Finally, bone density at the fracture line increased significantly over time in both groups (*p* < 0.001*), however no significant difference was noted comparing both groups together (*p* = 0.37).

**Conclusions:**

The addition of guided botulinum toxin A to the closed reduction protocols for subcondylar fractures significantly reduces postoperative pain and operative duration while optimizing alignment accuracy and functional mobility recovery. Given that bone healing remains uncompromised, this approach serves as an efficient, reliable alternative that directly improves patient outcomes.

**Trial status:**

Completed.

**Trial registration:**

The study was initially designed and ethically approved as an institutional clinical investigation. Trial registration was subsequently undertaken upon recognition of the importance of public trial registration in promoting transparency and facilitating dissemination of the study findings. The study protocol, eligibility criteria, outcome measures, and statistical analysis plan were defined prior to patient enrollment and remained unchanged following registration. The study was retrospectively registered with ClinicalTrials.gov on January 28, 2026 (NCT07374341).

## Introduction

The management of condylar and subcondylar fractures remains challenging, particularly when closed treatment is selected. Intermaxillary fixation (IMF) remains an established component of closed treatment, although the optimal duration of immobilization remains variable among treatment protocols. While shorter periods of IMF followed by early functional rehabilitation have increasingly been advocated, periods of 4–6 weeks have also been described for the conservative management of selected condylar and subcondylar fractures [1, 2]. Prolonged immobilization, however, may compromise early mandibular function and patient comfort, highlighting the need for strategies that may facilitate earlier functional rehabilitation while maintaining satisfactory fracture healing and occlusal stability [1–3].

Displacement of a fractured mandibular condylar or subcondylar segment is influenced by two principal forces: the initial traumatic force and the subsequent contraction of the attached masticatory muscles, particularly the masseter, temporalis, and lateral pterygoid muscles. Even when the fracture is initially undisplaced, continued muscular contraction may contribute to secondary displacement of the fracture segments during the healing period [4, 5]. Therefore, controlling these contractile forces may help maintain the position achieved by initial fracture reduction and preserve structural stability throughout healing, thereby potentially improving functional recovery and quality of life [4].

Accordingly, temporary reduction of masticatory muscle activity using botulinum toxin A (BTX-A) has been proposed to reduce the contractile forces acting on the fractured segment [4, 5]. Canter et al. were among the first to clinically investigate this concept, reporting the use of botulinum toxin A to temporarily reduce masticatory muscle activity and facilitate closed management of condylar fractures [4]. Although botulinum toxin type A (BTX-A) is generally considered safe and well tolerated, adverse effects can occur, including transient local injection-site reactions and unintended diffusion to adjacent structures, while systemic or hypersensitivity reactions are rare. Appropriate patient selection, dose optimization, accurate anatomical localization, and meticulous injection techniques are essential to minimize these risks, while prompt recognition allows appropriate symptomatic or medical management when complications occur [6, 7]. Over the years, the clinical utility of botulinum toxin A (BTX-A) has expanded to encompass a diverse range of medical indications involving skeletal muscle spasm or hyperactivity, including cerebral palsy, facial dystonias, and strabismus [4].

It has also been reported to reduce displacement of zygomatic body fractures by attenuating masseter muscle pull before surgery, resulting in successful preservation of the reduced zygomatic body segments, fewer operative procedures, and a reduced number of fixation plates required during surgery [8]. Furthermore, early evidence from Canter et al. demonstrated that temporary muscular paralysis facilitates the closed reduction of condylar and subcondylar fractures by counteracting the contractile forces exerted by the masticatory muscles [4].

Given the anatomical depth of the lateral pterygoid muscle and its proximity to critical neurovascular structures, particularly the maxillary artery, ultrasound (US) guidance provides real-time visualization of the target muscle and adjacent structures, facilitating accurate injection and reducing the risk of inadvertent vascular injury [9–11]. Al-Mahmoudy et al. investigated ultrasound-guided botulinum toxin A (BTX-A) injection as an adjunct to the closed management of condylar and subcondylar fractures, primarily targeting the lateral pterygoid muscle. Their findings provided preliminary clinical and radiographic evidence supporting the feasibility of this approach [5]. However, their study did not include a randomized conventional-treatment control group and focused on lateral pterygoid injection alone, without targeting the masseter or temporalis muscles. In the present study, we sought to evaluate the adjunctive effect of BTX-A within a randomized controlled design, while addressing the combined muscular forces involved in condylar fracture management through targeting of the lateral pterygoid, masseter, and anterior temporalis muscles, with quantitative assessment of clinical, functional, and radiographic outcomes.

Hence, the present study aimed to evaluate the effect of ultrasound-guided botulinum toxin type A (BoNT-A) injection into the masticatory muscles on postoperative functional recovery in patients undergoing closed reduction and intermaxillary fixation (IMF) for unilateral condylar and subcondylar fractures, with Maximum interincisal opening (MIO) defined as the primary outcome, while occlusal status, postoperative pain, fracture alignment, and radiographic healing were evaluated as secondary outcomes. The study was undertaken in view of the limited evidence regarding the potential biomechanical role of (BoNT-A) in reducing muscular traction forces on the fractured condylar segment and thereby facilitating early functional rehabilitation.

The null hypothesis of this study is that the botulinum toxin injection into the Masticatory muscles to manage the condylar and high subcondylar fractures will yield no significant difference compared to the conventional closed reduction and IMF method that is performed without any injections.

## Materials and methods

### Study design and setting

This randomized controlled clinical trial used a 1:1 allocation ratio and was conducted between June 2024 and March 2026, where patient recruitment was between June 2024 until September 2025, with follow up completed in March 2026. The study evaluated 16 participants presenting with unilateral subcondylar fractures, who were randomly distributed into two equal groups: 8 in the study group (A) receiving BTX injection along closed reduction with IMF, and 8 in the conventional control group (B) receiving the closed reduction and IMF without BTX injection, recruited from patients referred to and subsequently operated on within the Department of Oral and Maxillofacial Surgery, Faculty of Dentistry, Alexandria University. Ethical approval for this investigation was granted by the institutional Research Ethics Committee (IRB No. 00010556 - IORG 0008839), in strict accordance with the tenets of the Declaration of Helsinki [12]. Additionally, the clinical trial was retrospectively registered at ClinicalTrials.gov (NCT07374341), and it was conducted and reported in accordance with the CONSORT 2010 Statement for reporting parallel-group randomized controlled trials[13].

### Study participants

The current study included 16 patients aged 18 to 35 years old of both genders suffering unilateral subcondylar fracture. Patients were allocated into two groups, study group (A) included 8 patients who were managed by closed reduction and (IMF) accompanied by (BTX) injection to the masticatory muscles, and control group (B) included 8 patients treated conventionally by only closed reduction and (IMF).

### Sample size calculation, allocation and randomization

The sample size was calculated using MedCalc Statistical Software (MedCalc Software Ltd., Ostend, Belgium), assuming a two-sided significance level of 5% (α = 0.05), 80% statistical power, and equal allocation between the two study groups (1:1). The calculation was informed by previously published data for clinically relevant postoperative outcomes, including occlusion status, postoperative pain assessed using the Visual Analogue Scale (VAS), and maximum interincisal opening (MIO). Al-Mahmoudy et al [5], reported an unfavorable occlusion score in 1 of 10 patients (10%) treated with adjunct botulinum toxin injection, whereas Singh et al [14], reported unfavorable occlusion in 2 of 10 patients (20%) treated with closed reduction alone, corresponding to an absolute difference of 10% (95% CI: −34.7% to 51.8%). Postoperative pain scores, assessed using the Visual Analogue Scale (VAS), were 2.20 (1.03) and 2.30 (1.45) in the botulinum toxin and closed reduction groups, respectively, corresponding to a mean (SD) difference of 0.10 (1.24) and a 95% CI of −1.90 to 1.58. In addition, postoperative MIO was 4.20 (0.48) cm following botulinum toxin injection and 3.78 (0.26) cm following closed reduction, corresponding to a mean (SD) difference of 0.42 (0.37) cm and a 95% CI of 0.05 to 0.78 cm. Sample size calculations performed for all three outcomes yielded a minimum requirement of 7 patients per group. To compensate for potential loss to follow-up, the sample size was increased to 8 patients per group, resulting in a total sample size of 16 patients (8 per group) [15].

To conclude the sample size estimation, 16 patients will be recruited to decrease the margins of error and increase the accuracy of the study. Participants meeting the inclusion criteria were randomly assigned to one of the two study groups using a computer-generated randomization sequence. Allocation was performed by an independent investigator using the Sealed Envelope™ online randomization service (Sealed Envelope Ltd., London, UK), with equal block allocation to ensure balanced participant distribution between both groups. The allocation sequence was enclosed in sealed, opaque envelopes managed by an independent investigator, who was responsible to store the envelopes securely and open them sequentially only at the time of treatment [16].

### Blinding

This study was designed as a single-blinded randomized controlled clinical trial. Due to the inherent nature of the intervention, blinding of the operating surgeon and participants was not feasible, as the intervention group received ultrasound-guided botulinum toxin injections in addition to the conventional treatment protocol. However, the clinical and radiographic outcome assessments were performed by an independent examiner who was blinded to group allocation to minimize assessment bias.

#### Software

MedCalc Statistical Software version 19.0.5 (MedCalc Software bvba, Ostend, Belgium; https://www.medcalc.org; 2019)

### Eligibility criteria

#### Inclusion criteria [5, 17, 18]

The study included patients of both genders aged 18 to 35 years who presented with either isolated or associated unilateral extracapsular condylar or subcondylar fractures, or isolated intracapsular fractures. Eligible participants exhibited moderate condylar displacement (ranging from 10 to 45 degrees) or incomplete fractures. From a functional standpoint, candidates were required to demonstrate a reproducible occlusion. Additionally, adequate dental status was mandatory, defined as the retention and availability of more than half of the natural dentition.

#### Exclusion criteria [4, 5, 17, 18]

Patients presenting with bilateral condylar fractures or gross condylar displacement exceeding 45 degrees were excluded from the study. Exclusion criteria also applied to individuals with anatomic ramus height reduction of 2 mm, as well as those with unstable occlusion, defined as the presence of advanced periodontal disease or fewer than three functional teeth per quadrant. Furthermore, patients suffering from severe polytraumas were omitted. Finally, the study excluded individuals with motor neuron diseases or any relative contraindications to botulinum toxin (BTX) therapy, including pregnancy, lactation, known BTX allergies, or an active infection at the designated injection site.

## Intervention

### Prior to intervention, baseline pre-operative data was collected for all participants across both groups, divided into clinical and radiographic assessments.

#### Clinical assessments

The clinical follow-up was performed preoperatively and at 14 days, 6 weeks, and 3 months postoperatively. Maximum interincisal opening (MIO) and occlusal status was assessed preoperatively in both groups, with an additional (MIO) intraoperative assessment in the study group on the day of fracture reduction and intermaxillary fixation (IMF) application. IMF was removed at 14 days postoperatively in both groups to allow assessment of MIO and occlusion. MIO and occlusal status were assessed and compared between the two groups and with the corresponding preoperative results of each group at 14 days, 6 weeks, and 3 months postoperatively.

##### Maximum interincisal opening (MIO)

Unaided maximum interincisal opening (MIO), the primary outcome, was determined by using a ruler to measure in millimeter, the vertical distance between the maxillary and mandibular central incisal edges [5, 19]. This measurement was taken preoperatively, immediately following the BTX injection (for the study group), at 14 days, 6 weeks and 3 months postoperatively. At each designated time point, three independent readings were recorded and averaged to establish a mean value.

##### Occlusal assessment

Occlusal status was assessed clinically at each scheduled postoperative follow-up visit and compared with the patient’s pre-traumatic occlusion. Occlusal discrepancy was graded using a five-point ordinal scale previously described in the literature [2, 5, 14]: Occlusion assessment (1: Same to pre-traumatic, 2: Minor difference, 3: Functional malocclusion, 4: Needs occlusal correction, 5: Gross malocclusion). The assessment included evaluation of intercuspation, the presence of premature contacts, open bite or crossbite, and any clinically evident occlusal discrepancy or mandibular deviation on opening. The occlusal score was recorded for each patient at each assessment time point.

##### Preoperative pain assessment

Pain assessments were obtained across three key intervals: preoperatively, at the day of operation (fracture reduction), and subsequently at 14 days following the surgical operation, through a 10-point Visual Analogue Scale (VAS). (0–1 = None, 2–4 = Mild, 5–7 = Moderate, 8–10 = Severe) [20].

#### Radiographic assessments

**Fig. 1:**
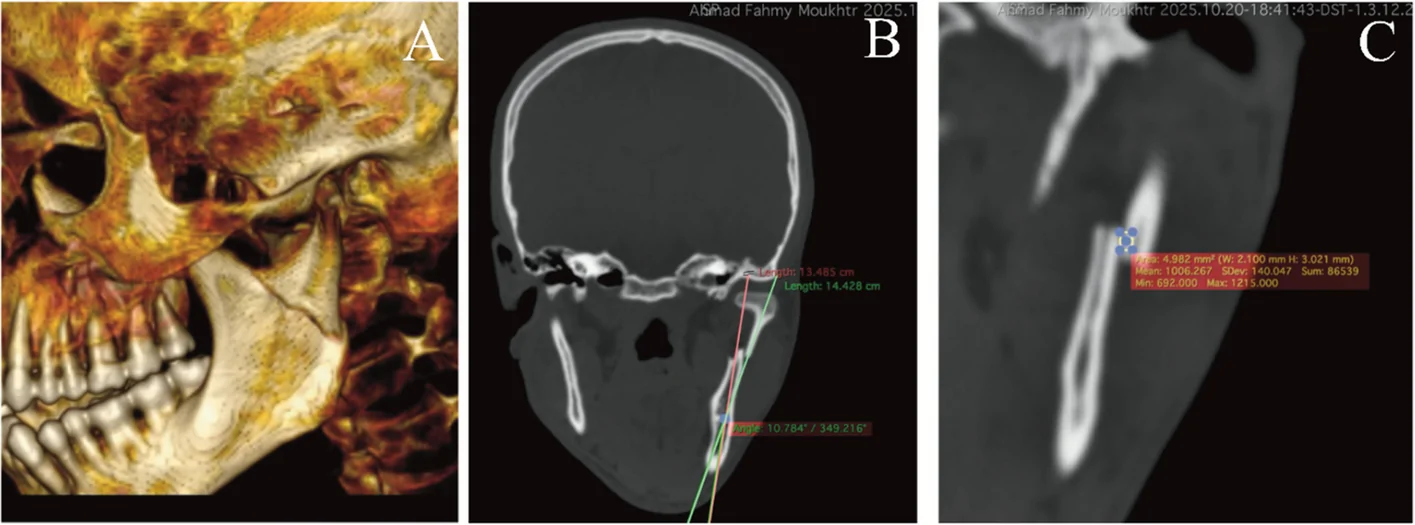
Preoperative computed tomography assessment demonstrating **A** 3D view of the subcondylar fracture. **B** Measurement of fracture angulation. **C** Bone density calculation at the fracture line

For both study and control groups, computed tomography (CT) scans were obtained pre-operatively, immediately after closed reduction and IMF application and lastly after a 6 month follow up, to examine the status of the subcondylar fracture, by measuring:

- The medio-lateral angulation in coronal CT view
  Calculating the angle between the longitudinal axis of the displaced subcondylar fragment and the longitudinal axis of the mandibular ramus [21, 22].
- The mean bone density at the fracture line (Fig. 1).

To quantify bone density adjacent to the fracture line, CT scans were analyzed and six readings at the fracture site were recorded and subsequently averaged to derive the mean bone density, expressed in Hounsfield units (HU) [23].

### Injection procedure for study group (A)

For study group (A), botulinum Toxin A (Botox; Allergan, Irvine, CA) injection was prepared by diluting the BTX vile with 2.0 cc saline, corresponding to a measurement of 5 Botox Units (BU) per 0.1 cc. After the injection of 2.0 cc of saline into botulinum toxin vial, it was 'gently' mixed not shook, (since shaking disrupts the interchain disulfide bonds between the heavy and light chains, leading to a reduced therapeutic effect), making it ready for use. [24]

Two to three days prior to the surgical intervention, botulinum toxin (BTX) was injected into the masticatory muscles on the fractured side, specifically targeting the lateral pterygoid, masseter, and temporalis. Topical anesthetic cream containing lidocaine 2.5% and prilocaine 2.5% (Pridocaine; Global Napi Pharmaceuticals, Egypt) was applied to the needle insertion site to anesthetize the skin and minimize needle insertion discomfort. The procedure was performed under ultrasound guidance utilizing a 25-gauge spinal needle. For the lateral pterygoid muscle, a dose of 10 to 20 units of BTX was administered, based on the dosing range reported by Al-Mahmoudy et al. [5].

**Fig. 2:**
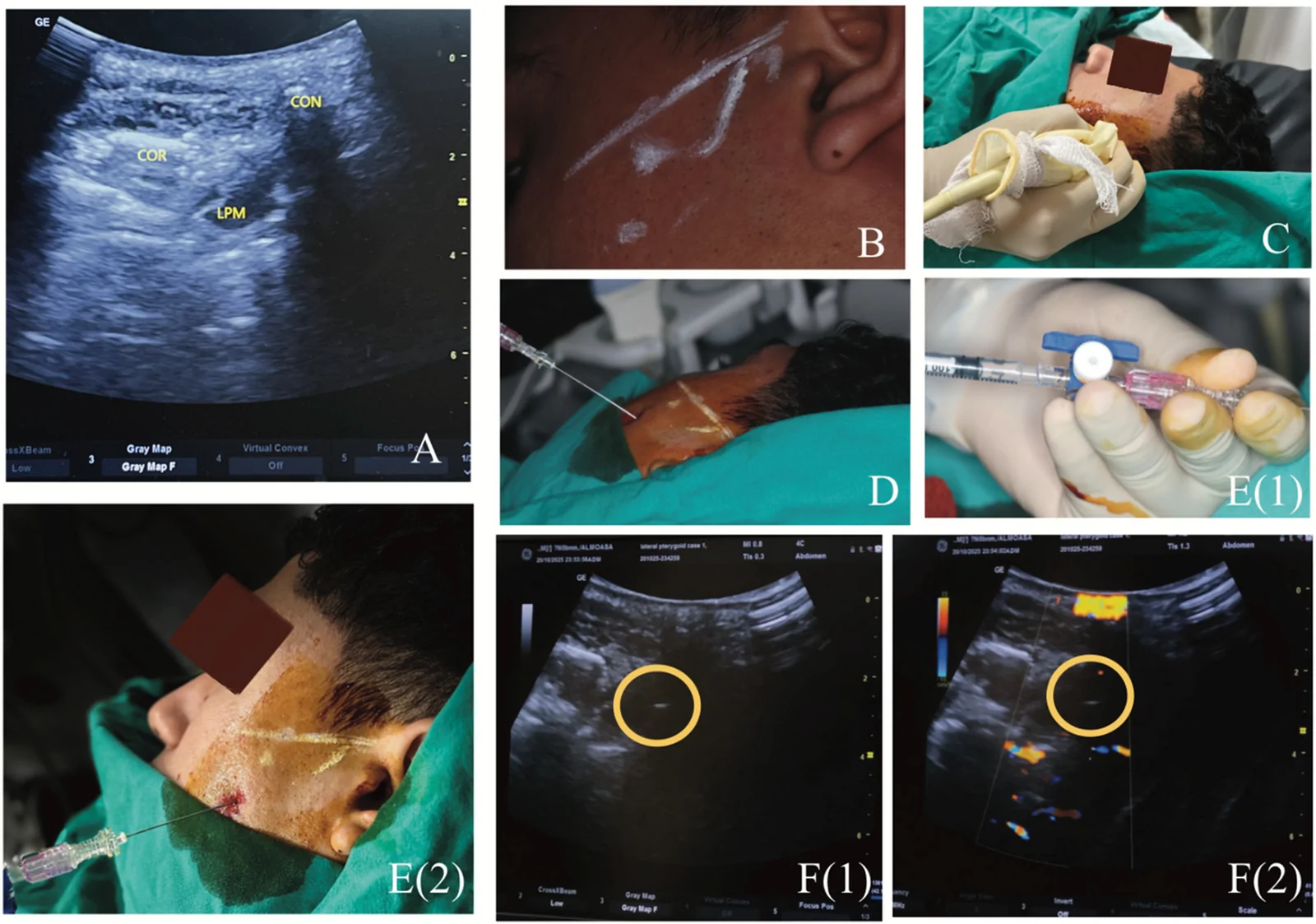
Ultrasound-guided lateral pterygoid muscle botulinum toxin administration in the study group. **A** Identification of the lateral pterygoid muscle (LPM) location using the ultrasound device, in between the coronoid process (COR) and the condyle (CON). **B** The demarcation of the extraoral landmarks for the needle insertion using a white face pencil. **C** After disinfection of the area, ultrasound probe used to relocate the LPM to insert the needle. **D** The spinal needle inserted at adequate depth reaching the LPM. (E1,2) Botulinum toxin injection into the LPM. (F1) The needle appears as an echogenic focus within the target musculature. (F2) Doppler mode is activated, showing far vasculature proximity. Written informed consent was obtained for the publication of these images

Correct intramuscular positioning was verified via ultrasound visualization, to access the LPM, the needle was advanced in an anteroinferior-to-posterosuperior trajectory [10], where the needle tip appeared on the screen as a distinct echogenic focus within the muscle belly [5]. (Fig. 2).

**Fig. 3:**
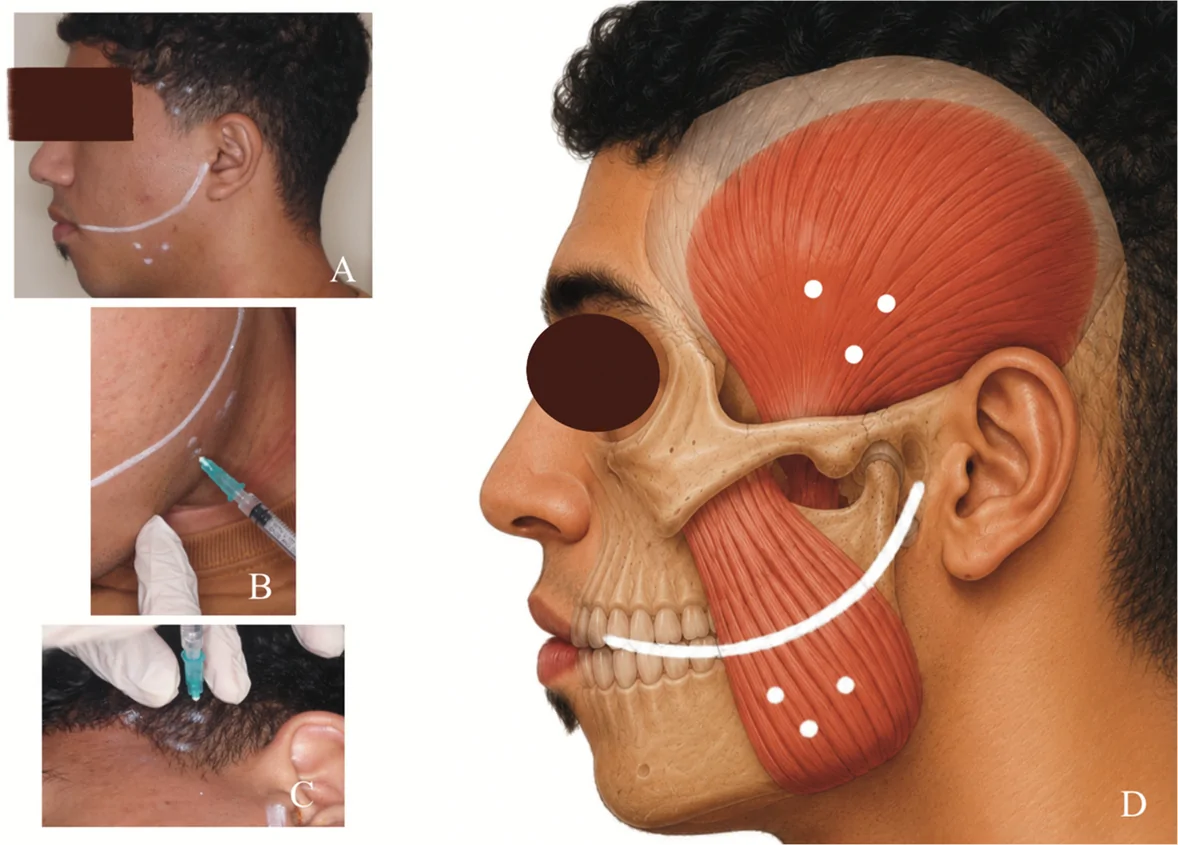
Botulinum toxin injection for the temporalis and masseter muscles in the study group. **A** Injection sites for botulinum toxin A administration within the masseter and temporalis musculature. **B** The injection of the masseter muscle. **C** The injection of the temporalis muscle. **D** Facial illustrative diagram representing the points of needle placements corresponding to the muscles. Written informed consent was obtained for the publication of these images

Additionally, the masseter muscle and the anterior fibers of the temporalis each received a transcutaneous injection of 30 IU of the toxin based on the protocol described by Canter et al. [4]. Participants were instructed to clench their teeth to allow identification and marking of the most prominent areas of the contracted muscle bellies, in order to distribute the points of injection upon them [25]. (Fig. 3).

Given that BTX diffusion is restricted to roughly 1 cm per site, an insufficient number of injection points risks leaving areas of the muscles unaffected, therefore, the calculated units were divided among multiple target sites to ensure an effective clinical response across the masticatory muscles [26].

**Fig. 4:**
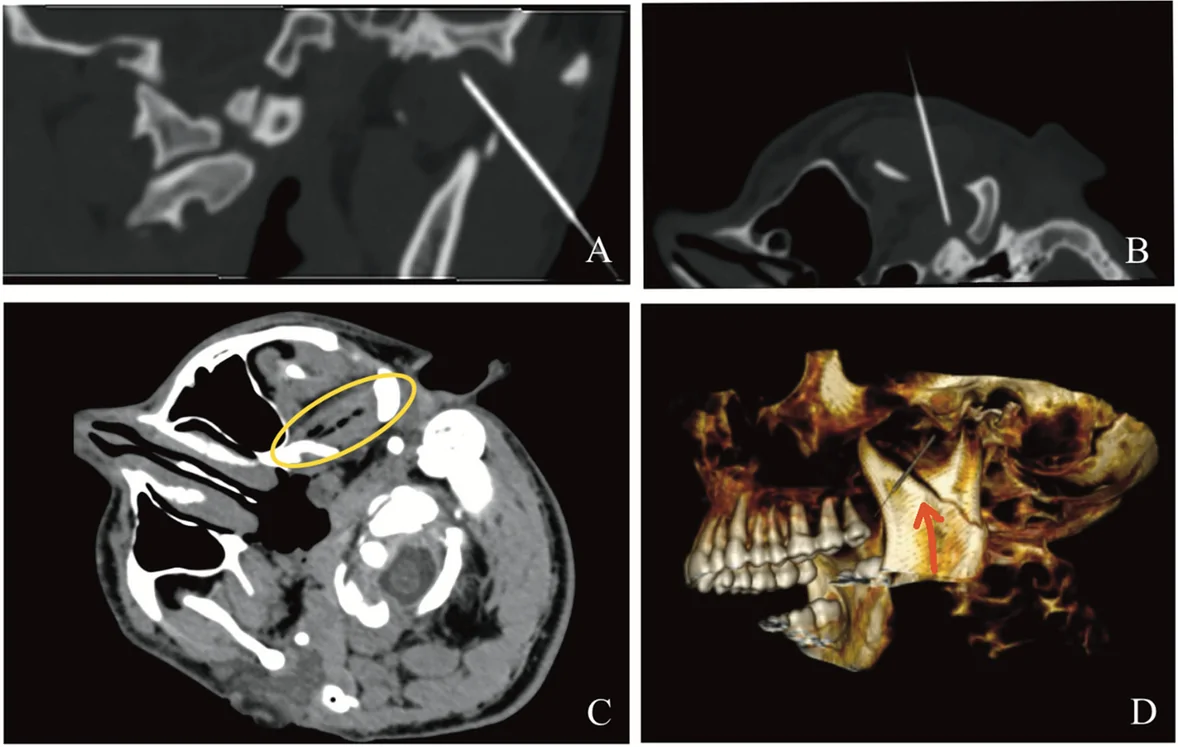
Computed Tomography scan was performed in the pilot study under ultrasound guidance to confirm the accuracy of ultrasound based localization in determination of the LPM location. **A** Sagittal view showing the needle insertion into the LPM. **B** Coronal view showing the needle insertion. **C** Black dots in the LPM indicate the precise spread of the botulinum toxin inside the LPM. **D** A 3D view of the needle insertion into the LPM

In addition, a pilot study was conducted to standardize the spinal needle insertion path under ultrasound guidance. The precision of the injection vector into the lateral pterygoid muscle (LPM) was cross verified using a confirmatory CT scan, thereby establishing a reproducible anatomical protocol for the remainder of the study group participants. (Fig. 4).

Although hypersensitivity reactions to botulinum toxin are rare, intra-dermal BTX sensitivity test was performed prior to the muscular injection by injecting 0.1 mL of toxin in the right or left forearm directly under the skin. The forearm skin was examined after 10 min, for any signs of hypersensitivity represented as erythematous edematous plaques, cutaneous rash or urticaria [7]. In the event of skin hypersensitivity reactions or systemic symptoms, such as nausea or flu-like manifestations, appropriate medical intervention was provided with corticosteroids and antihistamines [6, 7].

### Surgical intervention for both groups [5, 27]

**Fig. 5:**
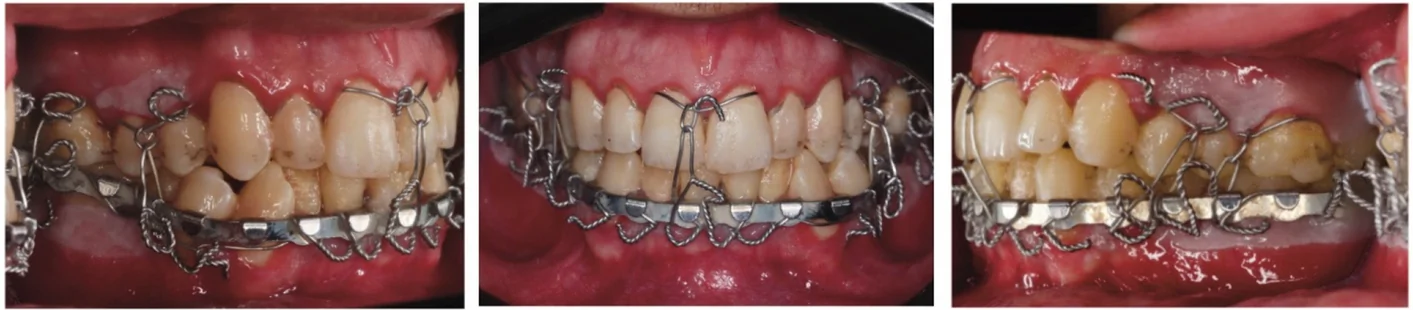
Closed reduction and intermaxillary fixation procedure using arch bar and eyelets

All patients across both groups underwent fracture management via closed reduction and intermaxillary fixation (IMF) under local anesthesia (Fig. 5). For patients in the study group, the IMF procedure was intentionally scheduled three days following the botulinum toxin injection, to allow the necessary 24 to 72 h for the neurotoxin to take clinical effect [4, 5]. To ensure a fair comparison, IMF for the control group was also performed three days after the participants’ enrollment.

For every case in the trial, the exact operative duration required to perform fracture reduction and intermaxillary fixation was recorded. Both groups underwent an initial 14-day period of intermaxillary fixation (IMF). At 14 days postoperatively, IMF was temporarily released in both groups to permit standardized clinical assessment of maximum interincisal opening (MIO) and occlusion [4, 5].Occlusion was assessed by comparing the postoperative occlusal relationship with the patient’s pre-traumatic occlusion. If satisfactory occlusion was achieved, no further IMF was required. If a residual occlusal discrepancy was identified, IMF was reapplied and maintained for the remaining 4 weeks, completing the conventional 6-week immobilization period according to the institutional protocol [1, 28]. Thus, the 14-day postoperative assessment was performed in both groups following an equivalent initial period of IMF, while the need for subsequent IMF was determined according to the occlusal findings at that assessment.

### Postoperative care

Patients of both groups were kept on a soft, fluid, high protein, high calorie diet for 4 weeks postoperatively, as for the postoperative medical regimen, it consisted of an initial course of intravenous cefotaxime (1 g every 12 hours) for the first 24 hours, followed by oral amoxicillin/clavulanate (1 g sachets twice daily) for the subsequent 5 days to have an adequate prophylactic coverage against infection. Concurrently, patients were prescribed metronidazole (500 mg syrup, 5 mL every 8 hours) and alpha-amylase (100 mg syrup, 5 mL every 8 hours) as an anti-inflammatory agent, both maintained for a 5-day duration. Analgesic and anti-inflammatory coverage was completed with diclofenac potassium (50 mg every 8 hours) for 5 days [28].

Patients were followed up twice weekly for the first two weeks, biweekly for the next two months, and then monthly up to 6 months postoperatively [5].

#### Assessment

Baseline clinical and radiographic assessments were performed preoperatively for all participants. Maximum interincisal opening (MIO) was designated as the primary outcome measure and was assessed preoperatively in both the study and control groups. In the study group, MIO was additionally assessed immediately after the BTX-A injection. Subsequent postoperative assessments of MIO were performed at 14 days, 6 weeks, and 3 months in both groups.

Secondary outcome measures included postoperative pain, occlusal status, operative duration, and radiographic parameters. Pain was assessed on the day of fracture reduction and at 14 days postoperatively, whereas occlusal status was evaluated preoperatively and at 14 days, 6 weeks, and 3 months postoperatively. The operative duration required for fracture reduction and IMF application was recorded for each patient. Radiographic assessment included evaluation of the mediolateral angulation of the fractured segment and bone density at the fracture line. These parameters were assessed immediately postoperatively and at 6 months postoperatively to evaluate fracture alignment and radiographic healing, respectively.

#### Statistical analysis

Statistical analysis was performed using IBM Statistical Package for the Social Sciences (SPSS) for Windows (Version 26.0). Continuous data were assessed for normality using descriptive statistics, histograms, Q–Q plots, and the Shapiro–Wilk test. Normally distributed quantitative data were expressed as mean ± standard deviation (SD), while non-normally distributed variables were presented as median and interquartile range (IQR).

For normally distributed data, comparisons between the two groups at each time point were performed using the independent samples t-test. Within-group comparisons across different follow-up periods were assessed using repeated measures analysis of variance (RM-ANOVA), followed by Bonferroni post-hoc correction for pairwise comparisons when applicable.

To evaluate the combined effects of treatment group and time, two-way repeated measures ANOVA were performed, including assessment of interaction effects between group and time. Adjusted means and standard errors (SE) with corresponding 95% confidence intervals (CI) were reported.

For non-normally distributed data (pain scores), comparisons between groups were performed using the Mann–Whitney U test, while within-group comparisons across time points were analyzed using the Friedman test, followed by Bonferroni-adjusted post-hoc pairwise comparisons.

A *p*-value < 0.05 was considered statistically significant.

## Results

Sixteen patients with unilateral subcondylar fractures were randomized equally into study and control groups (*n* = 8 each), with all participants completing the final analysis. Baseline demographics were highly comparable, showing no significant differences in age (24.88 ± 5.08 vs. 26.75 ± 5.95 years, *P* = 0.509) or gender distribution (*P* = 0.99; Table 1). Furthermore, no adverse events or treatment-related complications occurred in either group during the study period.

**Table 1:** Baseline demographic characteristics of the study participants

Variable	Study Group (*n* = 8) with BTX injection	Control Group (*n* = 8) Without BTX injection	*P*-value
Age (years), Mean ± SD	24.88 ± 5.08	26.75 ± 5.95	0.509
Gender, n (%)			0.99
Male	6 (75.0%)	5 (62.5%)	
Female	2 (25.0%)	3 (37.5%)	

**Fig. 6:**
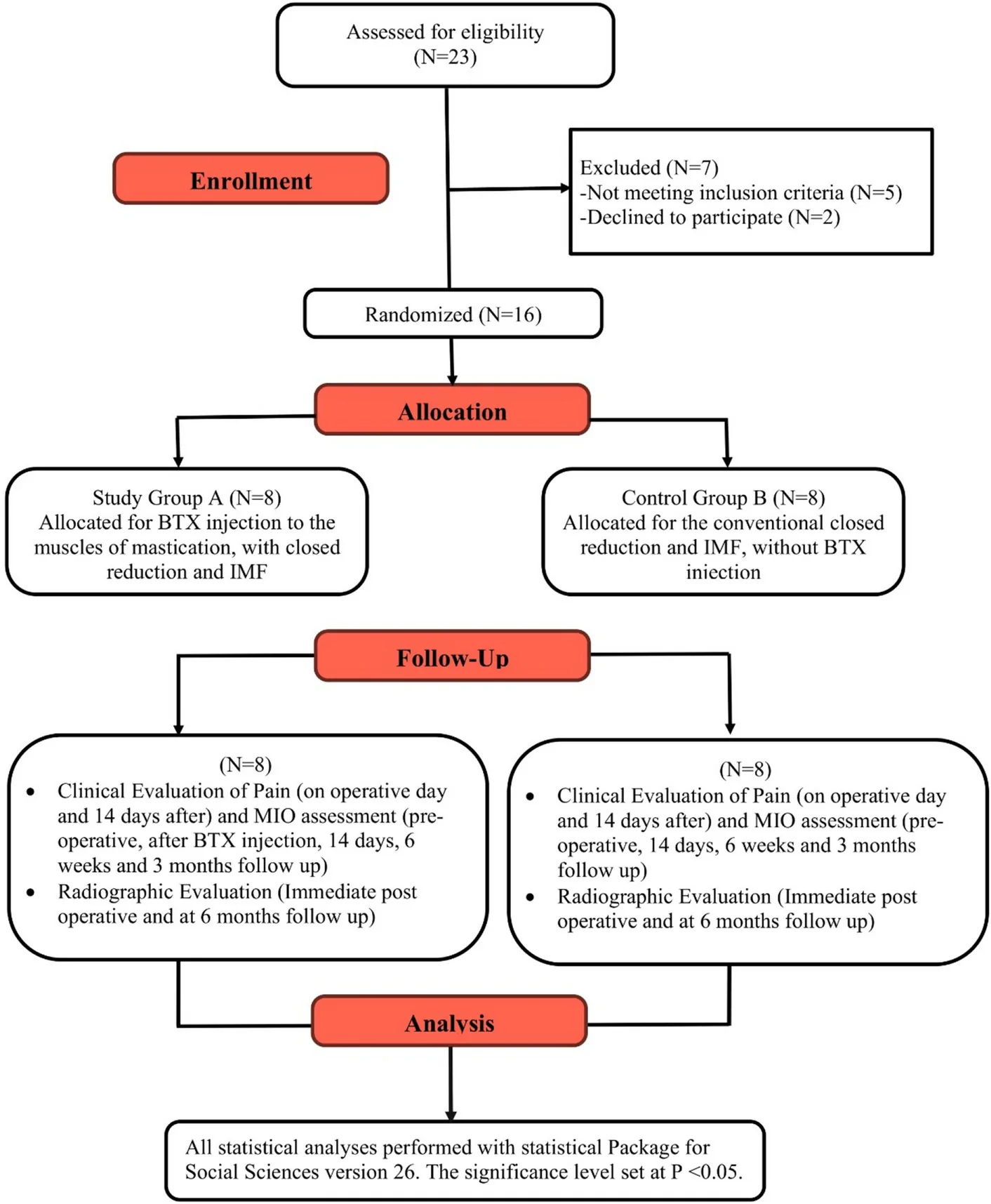
Flow diagram outlining the experimental phases and alignment with CONSORT guidelines

The CONSORT flow diagram illustrating the progression of participants throughout this randomized controlled clinical trial was noted in (Fig. 6).

### Clinical assessments

#### Mouth opening assessment (Fig. 7)

**Fig. 7:**
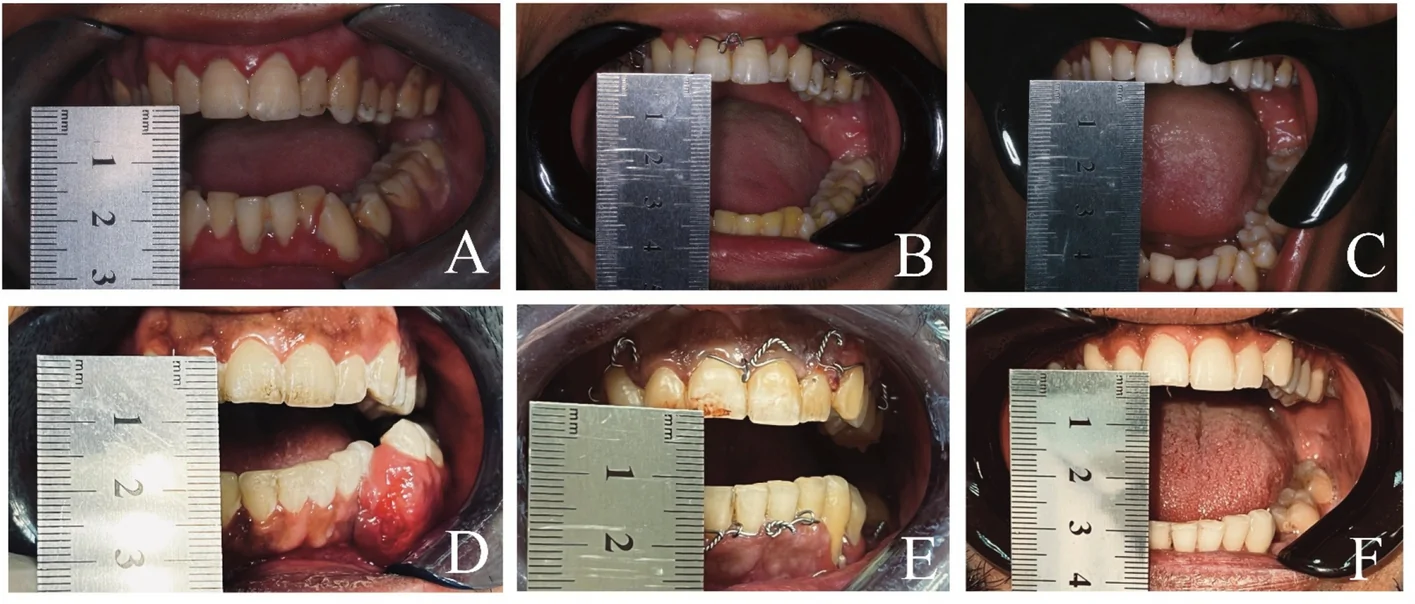
Clinical progression of maximal mouth opening during postoperative follow-up. **A** Corresponds to the preoperative interincisal distance for a participant from the study group. **B** Interincisal distance measurement after 14 days. **C** Interincisal distance measurement after 3 months. **D**, **E**, and **F** correspond to the same measurements for a participant from the control group, respectively. In the control-group participant, teeth 44 and 45 were severely affected by the initial trauma. Tooth 45 was completely avulsed with loss of its supporting alveolar bone and was extracted during the initial surgical procedure; therefore, it is absent in **E**. Tooth 44 was initially preserved because some supporting bone remained but was subsequently extracted during follow-up because of inadequate surrounding bony support and failure of satisfactory healing; therefore, it is absent in (**F**)

Patients in the study group demonstrated significantly greater mouth opening during the early postoperative follow-up periods, at 14 days postoperatively, mean mouth opening was significantly greater in the study group compared with the control group (31.44 ± 2.72 mm versus 14.46 ± 3.24 mm, respectively; *p* < 0.001*). Similarly, at 6 weeks postoperatively, the study group maintained significantly higher mouth opening values (38.02 ± 3.26 mm versus 17.88 ± 4.49 mm; *p* < 0.001*).

At 3 months postoperatively, both groups demonstrated substantial functional improvement, with no statistically significant difference observed between groups (*p* = 0.13). (Table 2).

**Table 2:** Comparison of mouth opening (mm) between the two groups

	**Study**	**Control**	**Mean difference (95% CI)**	***P* value 1**
	**Mean (SD)**			
**Pre-operative**	16.25 (3.21)** a**	13.15 (2.84)** a**	−0.15, 6.35	0.06
**Immediate**	23.53 (2.11)** b**	-	-	-
**14 days**	31.44 (2.72)** c**	14.46 (3.24)** b**	19.98 (13.77, 20.18)	< 0.001*
**6 weeks**	38.02 (3.26)** d**	17.88 (4.49)** c**	20.15 (15.94, 24.36)	< 0.001*
**3 months**	43.13 (3.27)** e**	38.38 (7.73)** d**	4.75 (−1.91, 11.41)	0.13
*P* value 2	< 0.001*	< 0.001*		

*SD* Standard Deviation, *CI* Confidence Interval

*P* value 1: Comparison between the two groups using independent samples t-test

*P* value 2: Comparison between different timepoints within each group using RM-ANOVA

*statistically significant at *p*-value < 0.05

a-e: different letters denote significant differences between timepoints using Bonferroni correction

Two-way repeated measures ANOVA demonstrated statistically significant effects of both time and treatment group on mouth opening measurements (*p* < 0.001*). Furthermore, a statistically significant interaction effect between group and time was observed (*p* < 0.001*), presented in (Table 3).

**Table 3:** Effect of group and time on mouth opening

		**Adjusted mean (SE)**	**95% CI**	***P* value**
**Group**	**Study**	32.21 (1.25)	29.53, 34.89	< 0.001*
	**Control**	20.97 (1.25)	18.29, 23.65	
**Time**	**Pre-operative**	14.70 (0.76) **a**	13.08, 16.32	< 0.001*
	**14 days**	22.95 (0.75)** b**	21.35, 24.56	
	**6 weeks**	27.95 (0.98)** c**	25.85, 30.06	
	**3 months**	40.75 (1.48)** d**	37.57, 43.93	

*SE* Standard Error, *CI* Confidence Interval

*Statistically significant at *p*-value < 0.05

a-c: different letters denote significant differences between time points using Bonferroni correction

The interaction between group and region was statistically significant (*p* < 0.001)

#### Occlusal assessment

Occlusal status was assessed using the predefined five-point scoring system, with lower scores indicating closer correspondence to the pre-traumatic occlusion. Preoperatively, the occlusal status distributions were comparable between the study and control groups, with a median score of 2 (IQR, 2–3) in both groups (*p* = 1.00). At 14 days, the study group demonstrated significantly more favorable occlusal status than the control group, with median scores of 1 (IQR, 1–1) and 2 (IQR, 2–2.75), respectively (*p* = 0.007). In the study group, 7 of 8 patients (87.5%) had occlusal status identical to their pre-traumatic occlusion (score 1), compared with 1 of 8 patients (12.5%) in the control group. At 6 weeks, median occlusal status was 1 (IQR, 1–1) in the study group and 1 (IQR, 1–1.75) in the control group (*p* = 0.72), with no significant between-group difference. Similar findings were observed at 3 months, with median scores of 1 (IQR, 1–1) and 1 (IQR, 1–1.75), respectively (*p* = 0.72). Within-group analysis demonstrated significant changes in occlusal status over time in both the study and control groups (Friedman test, *p* < 0.001 for both groups). (Table 4).

**Table 4:** Comparison of occlusal status between the groups across time

		**Study**	**Control**	***P* value 1**
		**N (%)**		
**Pre-operative**	Score 1	1 (12.5%)	1 (12.5%)	**1.00**
	Score 2	4 (50%)	4 (50%)	
	Score 3	2 (25%)	2 (25%)	
	Score 4	1 (12.5%)	1 (12.5%)	
	Score 5	0 (0%)	0 (0%)	
	Median (IQR)	2 (2–3) a	2 (2–3) a	
**14 days**	Score 1	7 (87.5%)	1 (12.5%)	0.007*
	Score 2	1 (12.5%)	5 (62.5%)	
	Score 3	0 (0%)	1 (12.5%)	
	Score 4	0 (0%)	1 (12.5%)	
	Score 5	0 (0%)	0 (0%)	
	Median (IQR)	1 (1–1) b	2 (2–2.75) ab	
**6 weeks**	Score 1	7 (87.5%)	6 (75%)	**0.72**
	Score 2	1 (12.5%)	2 (25%)	
	Score 3	0 (0%)	0 (0%)	
	Score 4	0 (0%)	0 (0%)	
	Score 5	0 (0%)	0 (0%)	
	Median (IQR)	1 (1–1) b	1 (1–1.75) b	
**3 months**	Score 1	7 (87.5%)	6 (75%)	**0.72**
	Score 2	1 (12.5%)	2 (25%)	
	Score 3	0 (0%)	0 (0%)	
	Score 4	0 (0%)	0 (0%)	
	Score 5	0 (0%)	0 (0%)	
	Median (IQR)	1 (1–1) b	1 (1–1.75) b	
***P* value 2**		< 0.001*	< 0.001*	

IQR: Interquartile range

*P* value 1: Comparison between the two groups using Mann–Whitney U test

*P* value 2: Comparison between different timepoints within each group using Friedman test

a-b: different letters denote significant differences between groups using Bonferroni correction

*statistically significant at *p*-value < 0.05

Bonferroni-adjusted post-hoc comparisons (Table 5), showed significant improvement from the preoperative assessment to 14 days, 6 weeks, and 3 months in the study group (*p* = 0.04 for each comparison), whereas in the control group, significant improvement was observed from the preoperative assessment to 6 weeks and 3 months (*p* = 0.02 for each comparison).

**Table 5:** Post-hoc comparison of occlusal status between timepoints with each group

Timepoint	Compared to	Study	Control
		***P* value**	
**Pre-operative**	**14 days**	0.04*	1.00
	**6 weeks**	0.04*	0.02*
	**3 months**	0.04*	0.02*
**14 days**	**6 weeks**	1.00	0.07
	**3 months**	1.00	0.07
**6 weeks**	**3 months**	1.00	1.00

*statistically significant at *p*-value < 0.05 (Bonferroni corrected)

#### Pain assessment

Preoperatively, both groups demonstrated comparable pain levels with no statistically significant difference (*p* = 1.00). On operation day, the study group demonstrated significantly lower pain scores compared with the control group, where the Mean VAS scores corresponded to 4.63 ± 1.19 in the study group compared to 8.63 ± 0.92 in the control group (*p* < 0.001*).

At 14 days postoperatively, pain scores continued to significantly improve in the study group compared with the control, as Mean VAS scores were 1.50 ± 0.93 in the test group and 6.63 ± 1.41 in the control group (*p* < 0.001*).

Within-group comparisons using the Friedman test demonstrated statistically significant reductions in pain scores across follow-up periods in both groups (study group: *p* < 0.001; control group: *p* = 0.001). (Table 6).

**Table 6:** Comparison of pain (VAS) between the two groups

		**Study**	**Control**	**Mean difference (95% CI)**	***P* value 1**
**Pre-operative**	**Mean (SD)**	8.75 (1.04) **a**	8.75 (1.04)** a**	0.00 (−1.11, 1.11)	1.00
	**Median (IQR)**	9.00 (8.00, 9.75)	9.00 (8.00, 9.75)		
**Operative**	**Mean (SD)**	4.63 (1.19)** b**	8.63 (0.92)** a**	−4.00 (−5.14, −2.86)	< 0.001*
	**Median (IQR)**	5.00 (3.25, 5.75)	9.00 (8.00, 9.00)		
**14 days**	**Mean (SD)**	1.50 (0.93)** c**	6.63 (1.41)** b**	−5.13 (−6.40, −3.85)	< 0.001*
	**Median (IQR)**	1.50 (1.00, 2.00)	6.50 (2.25, 7.75)		
*P* value 2		< 0.001*	0.001*		

*SD* Standard Deviation, *CI* Confidence Interval

*P* value 1: Comparison between the two groups using Mann–Whitney U test

*P* value 2: Comparison between different timepoints within each group using Friedman test

*statistically significant at *p*-value < 0.05

a-c: different letters denote significant differences between time points using Bonferroni correction

Bonferroni-adjusted post-hoc analysis demonstrated significant reductions between all evaluated time intervals in the study group, while the control group demonstrated delayed postoperative pain improvement, evident in (Table 7).

**Table 7:** Post-hoc comparisons of pain (VAS) across time within the two groups

Timepoint	Compared to	Study	Control
		**P value**	
**Pre-operative**	**Immediate**	0.04*	1.00
	**14 days**	< 0.001*	0.02*
**Operative**	**14 days**	0.04*	0.04*

*statistically significant using Bonferroni corrected *p*-value

## Operative time needed for closed reduction and intermaxillary fixation (Fig. 8)

**Fig. 8:**
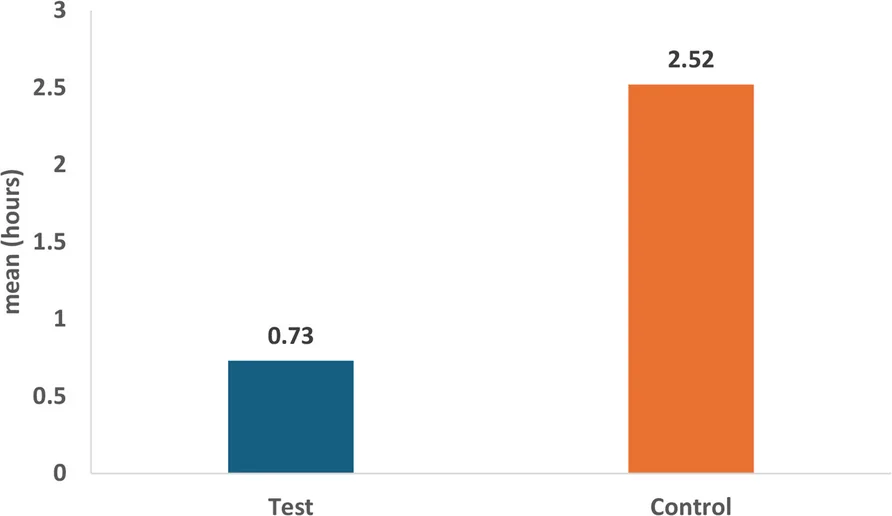
Bar chart representing operative time in hours in both study and control groups

The mean operative duration required for intermaxillary fixation application was significantly shorter in the study group compared with the control group, shown in (Table 8), as the average operative time in the study group was 43.77 ± 8.77 min, compared with 151.45 ± 18.71 min in the control group (*p* < 0.001*).

**Table 8:** Comparison of operative time between the two groups

	**Study**	**Control**	**Mean difference** **(95% CI)**	***P* value**
	**Mean (SD)**			
**Seconds**	2626.13 (526.15)	9086.75 (112.82)	−6460.63 (−7438.34, −5482.92)	< 0.001*
**Minutes**	43.77 (8.77)	151.45 (18.71)	−107.68 (−123.97, −91.38)	< 0.001*
**Hours**	0.73 (0.15)	2.52 (0.31)	−1.79 (−2.07, −1.52)	< 0.001*

*SD* Standard Deviation, *CI* Confidence Interval

Independent samples t-test was used

*statistically significant at *p*-value < 0.05

### Radiographic assessments

#### Fracture medio-lateral angulation assessment (Fig. 9)

**Fig. 9:**
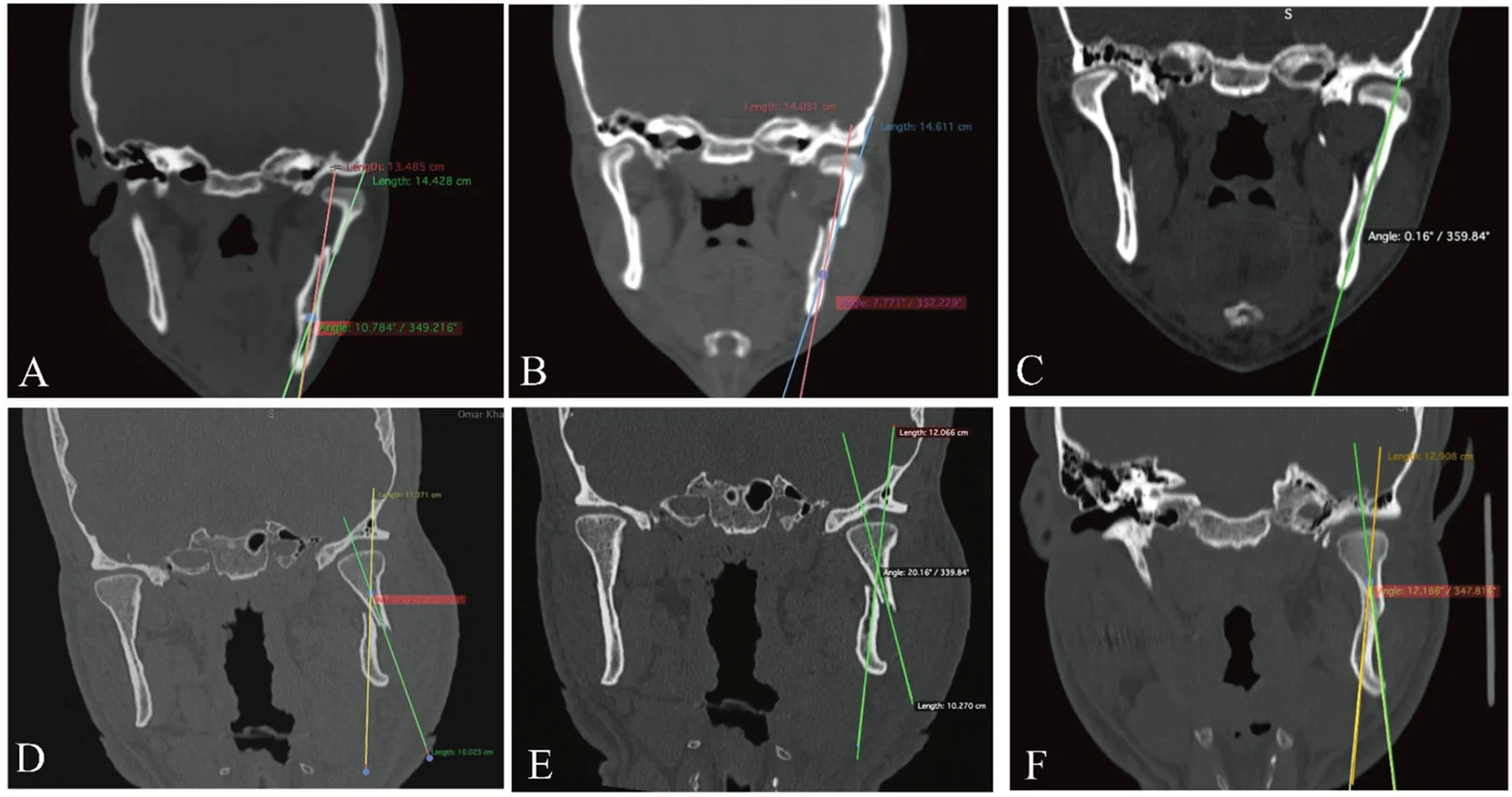
Radiographic comparison of fracture alignment (**A**–**C**) in a study-group patient and (**D**–**F**) in a control-group patient: **A**, **D** preoperatively, **B**, **E** immediately postoperatively, and (**C**, **F**) at 6 months following treatment

Immediately postoperatively, the study group demonstrated significantly lower angulation values compared with the control group (9.73 ± 1.63° versus 12.78 ± 3.04°, respectively; *p* = 0.03*). At the 6-month follow-up, the difference remained statistically significant, with the study group demonstrating near-anatomical alignment (0.42 ± 0.22°) compared with the control group (4.18 ± 3.50°) (*p* = 0.009*), presented in (Table 9).

**Table 9:** Comparison of angle between subcondylar fracture and the longitudinal axis of the ramus between the two groups

	**Study**	**Control**	**Mean difference (95% CI)**	***P* value 1**
	**Mean (SD)**			
**Pre-operative**	14.47 (2.30)** a**	16.20 (3.11)** a**	−1.73 (−5.01, 1.54)	0.28
**Immediate**	9.73 (1.63)** b**	12.78 (3.04)** b**	−3.05 (−5.67, −0.43)	0.03*
**6 months**	0.42 (0.22)** c**	4.18 (3.50)** c**	−3.76 (−6.42, −1.11)	0.009*
*P* value 2	< 0.001*	< 0.001*		

*SD* Standard Deviation, *CI* Confidence Interval

*P* value 1: Comparison between the two groups using independent samples t-test

*P* value 2: Comparison between different timepoints within each group using RM-ANOVA

*statistically significant at *p*-value < 0.05

a-c: different letters denote significant differences between time points using Bonferroni correction

Two-way repeated measures ANOVA demonstrated significant effects of both time (*p* < 0.001*) and treatment group (*p* = 0.02), while the interaction effect between group and time was not statistically significant (*p* = 0.34), noted in (Table 10).

**Table 10:** Effect of group and time on angle between subcondylar fracture and the longitudinal axis of the ramus

		**Adjusted mean (SE)**	**95% CI**	***P* value**
**Group**	**Study**	8.21 (0.75)	6.59, 9.82	0.02*
	**Control**	11.05 (0.75)	9.44, 12.66	
**Time**	**Pre-operative**	15.33 (0.76) **a**	13.70, 16.56	< 0.001*
	**Immediate**	11.25 (0.61)** b**	9.94, 12.56	
	**6 months**	2.30 (0.62)** c**	0.97, 3.63	

*SE* Standard Error, *CI* Confidence Interval

*statistically significant at *p*-value < 0.05

a-c: different letters denote significant differences between time points using Bonferroni correction

The interaction between group and region was not statistically significant (*p* = 0.34)

## Bone density at fracture line (Fig. 10)

**Fig. 10:**
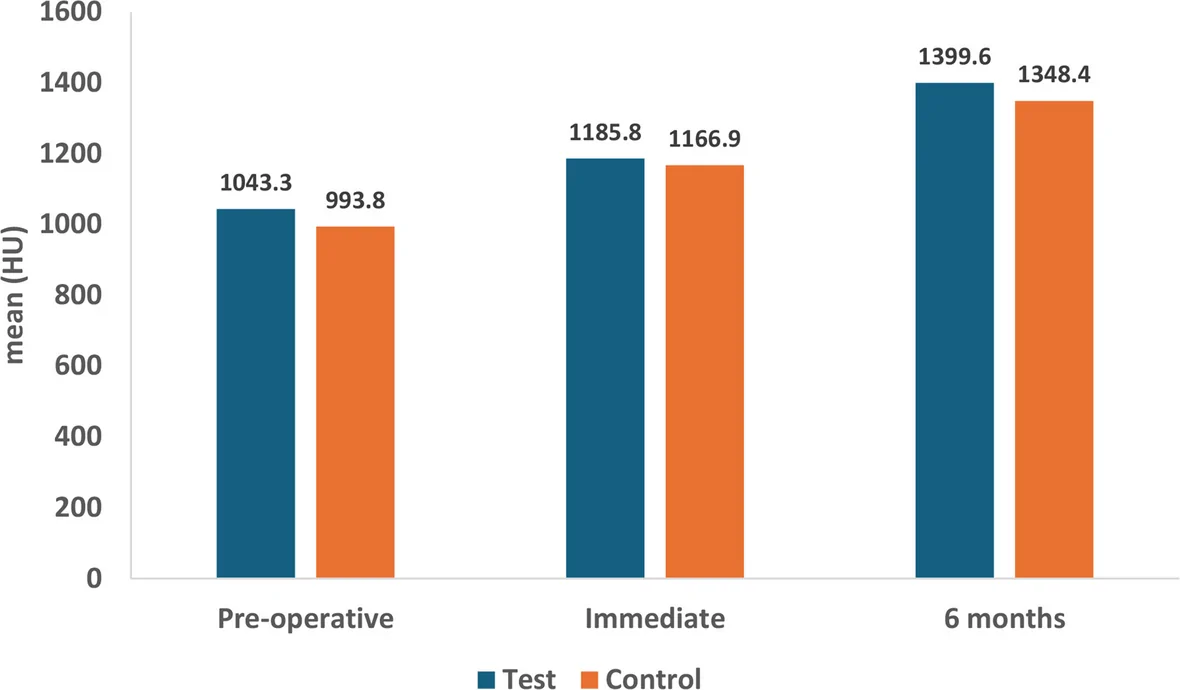
Bar chart showing bone density at fracture line (HU) in the two groups across time

In the study group, mean bone density increased from 1043.25 ± 45.12 HU preoperatively to 1399.63 ± 83.62 HU after 6 months. Similarly, the control group demonstrated an increase from 993.75 ± 72.10 HU preoperatively to 1348.38 ± 163.60 HU after 6 months. Within-group analysis revealed statistically significant increases across all follow-up periods in both groups (*p* < 0.001). However, intergroup comparisons showed no statistically significant differences at any time point (*p* > 0.05), noted in (Table 11).

**Table 11:** Comparison of bone density at fracture line (HU) between the two groups

	**Study**	**Control**	**Mean difference (95% CI)**	***P* value 1**
	**Mean (SD)**			
**Pre-operative**	1043.25 (45.12) **a**	993.75 (72.10)** a**	49.50 (−15.00, 114.00)	0.12
**Immediate**	1185.75 (24.42)** b**	1166.88 (131.59)** b**	18.87 (−91.57, 129.32)	0.70
**6 months**	1399.63 (83.62)** c**	1348.38 (163.60)** c**	51.25 (−88.07, 190.57)	0.44
*P* value 2	< 0.001*	< 0.001*		

*SD* Standard Deviation, *CI* Confidence Interval

*P* value 1: Comparison between the two groups using independent samples t-test

*P* value 2: Comparison between different timepoints within each group using RM-ANOVA

*statistically significant at *p*-value < 0.05

a-c: different letters denote significant differences between time points using Bonferroni correction

Two-way repeated measures ANOVA demonstrated a statistically significant effect of time on bone density values (*p* < 0.001), whereas the effect of study group was not statistically significant (*p* = 0.37). The interaction effect between group and time was also not statistically significant (*p* = 0.61), shown in (Table 12).

**Table 12:** Effect of group and time on bone density at fracture line (HU)

		**Adjusted mean (SE)**	**95% CI**	***P* value**
**Group**	**Study**	1209.54 (30.28)	1144.60, 1274.49	0.37
	**Control**	1169.67 (30.28)	1104.72, 1234.61	
**Time**	**Pre-operative**	1018.50 (15.04) **a**	986.25, 1050.75	< 0.001*
	**Immediate**	1176.31 (23.66)** b**	1125.57, 1227.06	
	**6 months**	1374.00 (32.48)** c**	1304.34, 1443.66	

SE: Standard Error, CI: Confidence Interval

*statistically significant at *p*-value < 0.05

a-c: different letters denote significant differences between time points using Bonferroni correction

The interaction between group and region was not statistically significant (*p* = 0.61)

## Discussion

Management of condylar and subcondylar fractures remains a subject of ongoing debate in maxillofacial trauma surgery, particularly when attempting to balance fracture stability, restoration of mandibular function, minimization of treatment-related morbidity, and preservation of patient quality of life. Although closed reduction with intermaxillary fixation (IMF) remains an established treatment modality, the duration of immobilization varies according to fracture characteristics, treatment protocols, and clinical practice, with periods of several weeks traditionally used to maintain fracture stability and occlusal relationships [3, 4, 14, 26]. However, prolonged immobilization may be associated with functional limitations and patient discomfort, highlighting the need for strategies that may permit earlier functional rehabilitation while maintaining satisfactory fracture stability.

The present study was designed around a biomechanical concept that has received relatively limited investigation in condylar fracture management: the potential influence of masticatory muscle activity on the stability of the fractured condylar segment during reduction and the subsequent healing period. While previous literature has acknowledged the displacement effect of the lateral pterygoid muscle in condylar fractures, few studies have comprehensively addressed the contribution of the lateral pterygoid, masseter, and temporalis muscles as dynamic forces that may influence fracture stability and functional recovery [4, 5, 26]. Accordingly, the rationale for incorporating botulinum toxin A (BTX-A) was not limited to symptomatic pain reduction but also involved temporary modulation of masticatory muscle activity around the fracture site, with the aim of reducing muscular traction and facilitating fracture reduction and subsequent functional rehabilitation [4, 8, 29].

The modulation of the muscular environment was reflected in the significantly greater mouth-opening values observed during the early postoperative period in the study group. At the 14-day assessment, the study group demonstrated significantly greater maximal mouth opening than the control group (31.44 ± 2.72 mm versus 14.46 ± 3.24 mm, respectively; p < 0.001). Importantly, both groups had undergone the same 14-day period of intermaxillary fixation before this assessment, providing comparable mechanical immobilization at the time of the early functional evaluation. The greater mouth opening observed in the study group may therefore be related to the temporary reduction in masticatory muscle activity following BTX-A administration, which may have reduced muscular resistance during the early stages of recovery [4, 5, 29]. This early improvement in mandibular mobility suggests that incorporating BTX-A into the treatment protocol may facilitate earlier functional rehabilitation while maintaining the principles of closed fracture management.

The pharmacological profile of BTX-A is also relevant when interpreting the temporal pattern of functional recovery. Following intramuscular administration, botulinum toxin type A (BTX-A) inhibits acetylcholine release at the neuromuscular junction, resulting in a progressive reduction in muscle activity. The clinical effects typically begin to emerge within several days after injection, although an onset of action may be observed as early as 24 h in some cases [30].The effects gradually develop over the following weeks and may persist for several months [30–32]. Thus, its neuromuscular effect extends beyond the initial period of immobilization and may continue to influence masticatory muscle activity during subsequent functional recovery. In the present study, the significantly greater maximal mouth opening observed at 14 days occurred after an equivalent initial 14-day period of IMF in both groups, suggesting an early functional advantage associated with BTX-A-assisted treatment.

Consistent with this early difference in MIO, occlusal status provided an additional clinical measure of functional recovery, reflecting an important outcome in the assessment of mandibular condylar fracture treatment [2, 14]. At 14 days, patients receiving BTX-A demonstrated more favorable occlusal status than those in the control group, with most patients in the BTX-A group achieving an occlusal relationship identical to their pre-traumatic occlusion. This early difference may reflect more effective early control of masticatory muscle forces following BTX-A administration [25, 32]. At later assessments, the observed functional differences should be considered in the context of the sustained pharmacological effect of BTX-A together with the subsequent duration of IMF, which was determined according to the occlusal findings at the 14-day assessment. Importantly, the between-group difference in occlusal status was no longer evident at 6 weeks or 3 months, with both groups demonstrating predominantly favorable occlusal relationships at the later assessments. By 3 months, the difference in maximal mouth opening between the two groups was no longer statistically significant (43.13 ± 3.27 mm versus 38.38 ± 7.73 mm; *p* = 0.13), suggesting that the observed functional benefit of the intervention was primarily related to earlier functional recovery rather than a persistent superiority in maximal mouth opening. Taken together, these findings suggest that BTX-A may facilitate earlier restoration of functional and occlusal recovery during the early postoperative period, while longer-term outcomes become comparable between treatment protocols.

The dosing strategy used in the present study was guided by previously described applications of BTX-A in fracture management. The 30 U doses administered to the masseter and anterior temporalis were based on the protocol described by Canter et al. [4], while the lateral pterygoid was selectively targeted under ultrasound guidance using 10–20 U, within the range reported by Al-Mahmoudy et al. [5]. Although lower doses to the temporalis have been reported in other clinical applications of BTX-A, the dose used in the present study was selected according to the fracture-management protocol and the specific therapeutic objective of transiently reducing masticatory muscle activity during fracture healing. These dosing approaches therefore reflect the muscle targets and clinical objectives of the present protocol rather than a standardized dose applicable across different indications [29].

In Study Group A, a notable finding was the early reduction in postoperative pain following BTX-A administration. This observation is consistent with previous clinical evidence suggesting that BTX-A may have analgesic effects in addition to its neuromuscular activity. Rady et al. reported significant improvement in pain and joint clicking following BTX-A injection into the lateral pterygoid muscle in patients with temporomandibular disc displacement [30], Similarly, Hamdy et al. reported lower postoperative pain scores in children undergoing lower-limb lengthening and deformity correction following BTX-A administration [33]. These findings provide clinical support for a potential analgesic role of BTX-A across different musculoskeletal conditions; however, differences in patient populations, treatment indications, and injection protocols should be considered when comparing these findings with the present study.

The lower postoperative pain scores observed in the present study may reflect the combined effects of reduced masticatory muscle activity and the potential antinociceptive properties of BTX-A. Botulinum toxin has been proposed to modulate nociceptive signaling through inhibition of peripheral neurotransmitter release and reduction of neurogenic inflammation [31, 34]. Nevertheless, the present study did not directly assess peripheral nociceptive mediators or neuromuscular activity. Therefore, the observed reduction in pain should be interpreted as a clinical finding associated with the BTX-A-assisted treatment protocol rather than as direct evidence of a specific analgesic mechanism.

Furthermore, previous studies have suggested that excessive muscular traction and spasm can complicate the manipulation of fractured segments and hinder accurate anatomical reduction. Canter et al. [4], demonstrated that botulinum toxin (BTX) can reduce these displacing muscular forces by decreasing masticatory muscle activity, thereby facilitating conservative management of mandibular condylar fractures. In the present study, the BTX-A-assisted treatment was associated with significantly improved fracture angulation at six months, with a mean angulation of 0.42 ± 0.22° in the study group compared with 4.18 ± 3.50° in the control group (*p* = 0.009*). This finding is consistent with the proposed role of BTX-A in reducing excessive masticatory muscle activity during the early management of condylar fractures respectively. This finding is consistent with the observations reported by Akbay et al. [25], who advocated using BTX to reduce counterproductive masticatory forces during the reduction procedure. This reduction in muscle spasm not only contributed to improved fracture alignment but also explained the significantly shorter operative duration, as the reduction process was facilitated and associated with less pain. Akbay et al. [25] similarly described the potential utility of BTX-A in reducing counterproductive masticatory forces during fracture management. However, because muscle activity was not directly measured in the present study, the observed improvement in fracture angulation should be interpreted as an association with the BTX-A-assisted treatment rather than direct evidence that reduced muscle activity was the sole mechanism responsible. The significantly shorter operative duration observed in the study group may likewise reflect the overall effect of the treatment protocol, including facilitated manipulation and reduction, rather than being attributed exclusively to BTX-A-mediated reduction of muscle spasm.

Both the study group (A) and control group (B) demonstrated comparable increases in bone density throughout the healing period, despite the shorter IMF duration in the study group. This finding is consistent with previous experimental evidence suggesting that botulinum toxin A does not necessarily impair fracture healing. Hao et al. [35] reported that local BTX-A-induced muscle atrophy may transiently affect fracture healing, whereas Tukel et al. [36] found no adverse effect of BTX-A administration on mandibular fracture healing. In the present study, the absence of a significant difference in bone density between the two groups suggests that the adjunctive use of BTX-A did not result in an evident detrimental effect on radiographic fracture healing. However, because the pharmacological effect of BTX-A may persist beyond the period of IMF and the present study did not directly quantify neuromuscular activity or chemodenervation, the observed bone-healing findings should be interpreted as clinical and radiographic observations rather than evidence of a specific duration-dependent muscular or biological effect of BTX-A.

Although IMF is intended to preserve fracture stability and maintain the interrelationship of the occlusal surfaces [1], prolonged immobilization may be associated with compromised oral hygiene, nutritional difficulties, temporomandibular joint stiffness, impaired speech, patient discomfort, and psychological distress [2, 37]. Botulinum toxin A has therefore been proposed as an adjunctive means of reducing masticatory muscle activity during closed fracture management. Through temporary chemodenervation, BTX-A may reduce excessive muscular contraction and the resultant displacing forces acting on the fractured condylar segment, potentially facilitating maintenance of fracture alignment during functional recovery [38]. Canter et al. [4] demonstrated that paralysis of the masticatory muscles facilitated closed treatment of condylar fractures and contributed to maintaining fracture alignment with minimal displacement. Similarly, Shilt et al. [38], reported the use of botulinum toxin A as a chemoprotective rehabilitation adjunct in proximal hamstring avulsion injuries, where reduction of counterproductive muscular forces permitted earlier controlled rehabilitation. In the present study, BTX-A was used as an adjunct to mechanical fixation with the aim of reducing muscular forces and facilitating earlier functional rehabilitation. The findings support the potential role of BTX-A-assisted management in facilitating earlier functional recovery while maintaining satisfactory fracture healing.

Due to the anatomical depth of the lateral pterygoid muscle and its proximity to critical vascular structures, particularly the maxillary artery, ultrasound guidance was used to facilitate accurate and safe injections. Al Mahmoudy et al. [5] highlighted the anatomical considerations associated with this region, while previous studies have demonstrated the feasibility of ultrasound-guided assessment and injection of the lateral pterygoid muscle [9]. In the present study, real-time ultrasound guidance, supplemented by Doppler imaging, enabled visualization of the target muscle and assessment of adjacent vascular structures during injection [10, 11]. This approach was therefore adopted to enhance procedural precision and minimize the potential risk of inadvertent vascular injury.

**Fig. 11:**
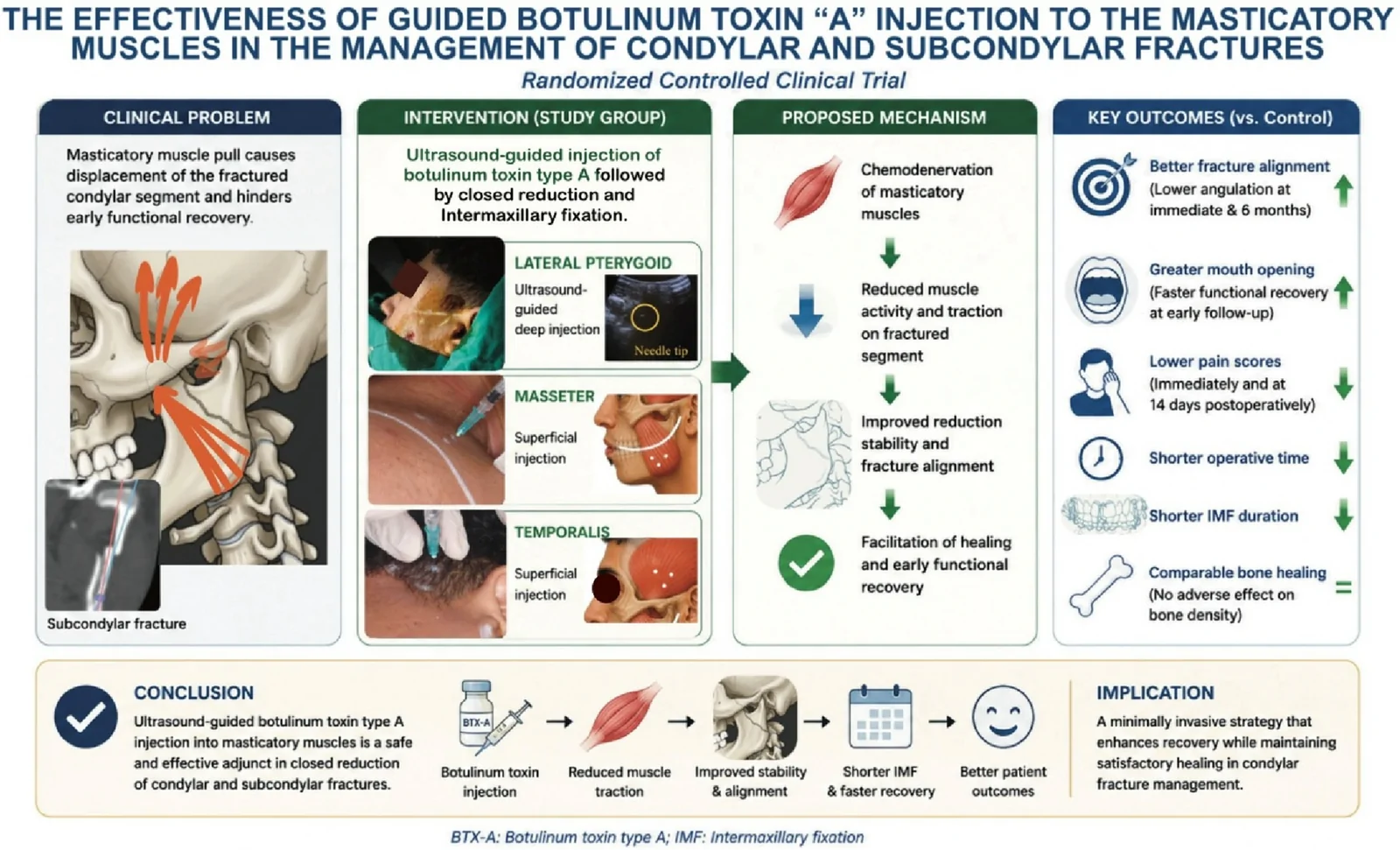
Shows a summary for the clinical trial in a form of a graphical representation of the biomechanical effect of ultrasound-guided botulinum toxin injection into the muscles of mastication during closed reduction management of condylar and subcondylar fractures, demonstrating reduced muscular traction, improved fracture alignment, shorter intermaxillary fixation duration, and enhanced postoperative rehabilitation

Collectively, the present findings suggest that botulinum toxin A may serve as a useful adjunct in the closed management of subcondylar fractures by reducing masticatory muscle activity and facilitating the management and functional rehabilitation of the fractured segment. In combination with conventional mechanical fixation, BTX-A-assisted treatment may provide a minimally invasive approach between conventional closed management and open surgical treatment, while potentially allowing earlier functional recovery [4, 25, 26]. The overall treatment protocol and study workflow are summarized in the graphical abstract (Fig. 11).

Nevertheless, this study has several limitations that should be noted. Although designed as a randomized controlled trial, the relatively small sample size may limit the generalizability of the data. Furthermore, the six-month follow-up window may not fully reflect long-term functional performance, temporomandibular joint adaptation, or late-stage skeletal remodeling. In terms of protocol, despite the advantages of ultrasound-guided botulinum toxin injections, the procedure is operator-dependent and relies on the clinician's experience. The high cost of botulinum toxin A also represents a notable clinical limitation. Lastly, because direct electromyographic assessment of the masticatory muscles was not performed, the biomechanical effects of the toxin were inferred from clinical and radiographic outcomes rather than objectively quantified.

## Conclusion

Within the limitations of this prospective randomized controlled clinical trial, ultrasound-guided botulinum toxin A injection into the masticatory muscles may serve as an effective adjunctive modality for the closed reduction of unilateral subcondylar fractures. Compared with the conventional protocol, this technique is associated with improved fracture alignment, earlier functional recovery, and reduces postoperative pain while significantly shortening both operative time and IMF durations. Most importantly, these clinical advantages were achieved without compromising primary bone healing outcomes. Thus, these findings indicate that temporary chemo-immobilization and modulation of masticatory muscle activity may represent a potentially useful minimally invasive strategy for optimizing patient-centered outcomes in subcondylar fracture management.

## Data Availability

To comply with institutional privacy protocols, the primary datasets evaluated throughout this investigation are restricted from public repositories. However, anonymized data can be obtained directly from the corresponding author upon receiving a justified request.

## Abbreviations

BTX-A: Botulinum Toxin A
BU: Botox Units
CON: Condyle *(used in anatomical/ultrasound figures)*
CONSORT: Consolidated Standards of Reporting Trials
COR: Coronoid Process *(used in anatomical/ultrasound figures)*
CT: Computed Tomography
HU: Hounsfield Units
IMF: Intermaxillary Fixation
LPM: Lateral Pterygoid Muscle
MIO: Maximum Interincisal Opening
US: Ultrasound
VAS: Visual Analogue Scale
